# Biomedical Applications of Graphene-Based Structures

**DOI:** 10.3390/nano8110944

**Published:** 2018-11-16

**Authors:** Krzysztof Tadyszak, Jacek K. Wychowaniec, Jagoda Litowczenko

**Affiliations:** 1 NanoBioMedical Centre, Adam Mickiewicz University, ul. Umultowska 85, PL61614 Poznań, Poland; jacek.wychowaniec@amu.edu.pl or jacek.wychowaniec@ucd.ie (J.K.W.); jagoda.litowczenko@amu.edu.pl (J.L.); 2 School of Chemistry, University College Dublin, Belfield, Dublin 4, Ireland; 3 Department of Molecular Virology, Faculty of Biology, Adam Mickiewicz University, Umultowska 89, PL61614 Poznań, Poland

**Keywords:** graphene, graphene oxide, nanomaterial toxicity, tissue engineering, regenerative medicine, nanostructured materials, anticancer therapies

## Abstract

Graphene and graphene oxide (GO) structures and their reduced forms, e.g., GO paper and partially or fully reduced three-dimensional (3D) aerogels, are at the forefront of materials design for extensive biomedical applications that allow for the proliferation and differentiation/maturation of cells, drug delivery, and anticancer therapies. Various viability tests that have been conducted in vitro on human cells and in vivo on mice reveal very promising results, which make graphene-based materials suitable for real-life applications. In this review, we will give an overview of the latest studies that utilize graphene-based structures and their composites in biological applications and show how the biomimetic behavior of these materials can be a step forward in bridging the gap between nature and synthetically designed graphene-based nanomaterials.

## 1. Introduction

**Figure 1:**
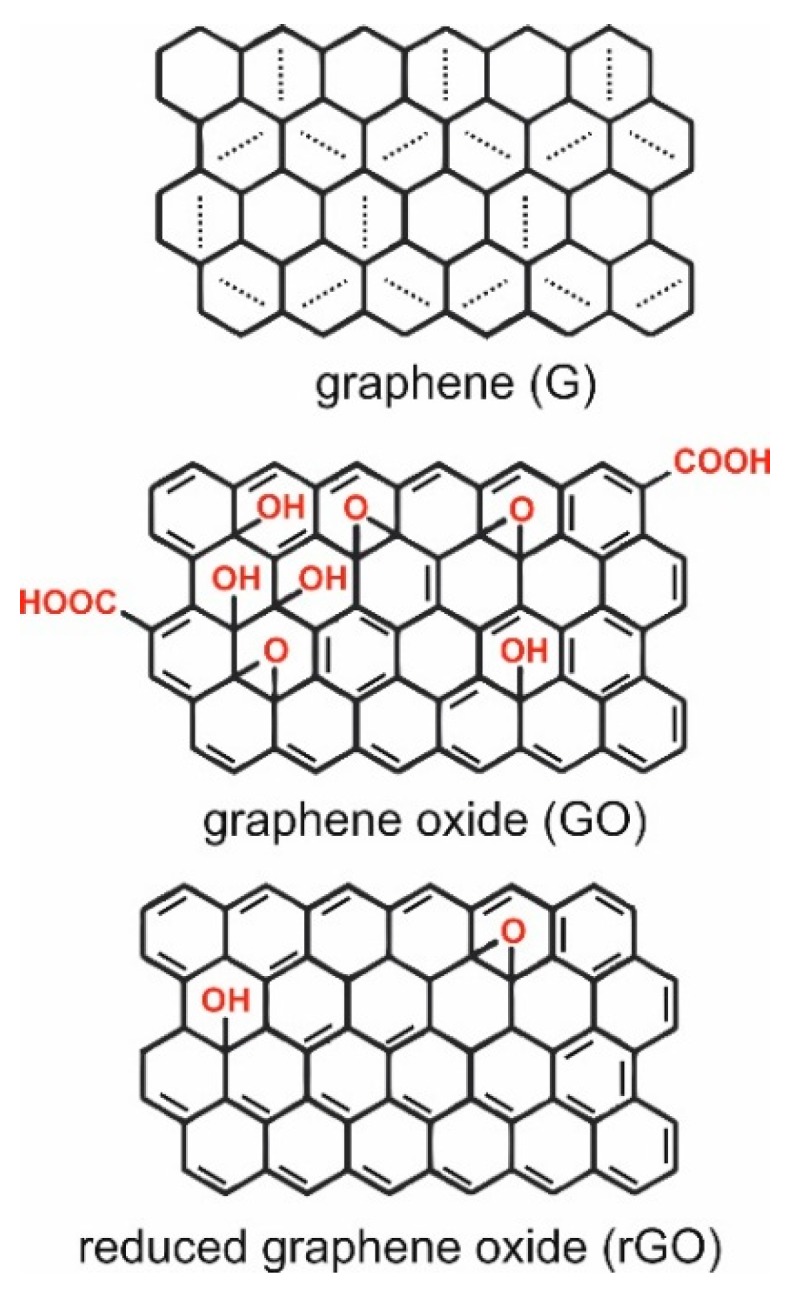
Structures of graphene (G), graphene oxide (GO), and reduced graphene oxide (rGO). According to the adaptive natural density partitioning analysis and the electron sharing indices, graphene is aromatic, but its aromaticity is different from the aromaticity in benzene. Aromaticity in graphene is local with two π-electrons located over every hexagon ring [8].

Carbon materials form one of the most abundant groups of materials, and are used in applications that range from electronics to biotechnology [1,2]. Not only do there exist many allotropic forms of carbons: diamond, graphene, fullerene, nanotube [3], but nowadays there are many allotropic forms of only graphene [4,5]. Graphene (G) is also known as a graphene layer, single-layer graphene, or monolayer graphene, and according to the most recent ISO standard (ISO/TS 80004-13:2017) it is a single layer of carbon atoms with each atom covalently bound to three neighbors in a honeycomb structure [6]. It can be best represented as a pure carbon monocrystalline graphitic sheet comprising a single layer of carbon atoms densely packed into a benzene-ring structure (Figure 1) [7]. The typical route by which one can obtain graphene is a top-down approach from graphite, which consists of graphene layers stacked parallel to each other in a three dimensional, crystalline, long-range order [6].

**Figure 2:**
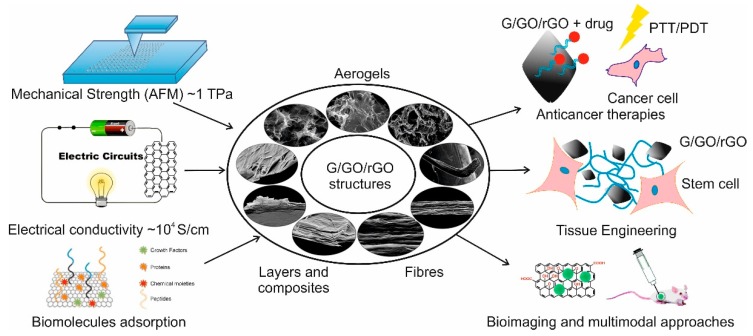
The scheme showing graphene (G), graphene oxide (GO), and reduced graphene oxide (rGO) structures (author’s original SEM images of GO paper, fibers, and aerogels), their excellent mechanical, electrical, and biological properties, and their typical uses in biomedical applications that are discussed in this review.

Graphene oxide (GO) is a chemically modified graphene that is typically prepared by oxidation and exfoliation of graphite-bearing oxygen functional groups, such as carboxyl (-OOH), hydroxyl (-OH), or epoxy (-O), on their basal planes and edges (Figure 1) with the modified Hummers’ method being the golden standard technique for its production [9,10]. Chemical, thermal, microwave, photo-chemical, photo-thermal, or microbial/bacterial treatments can be used on GO to reduce the oxygen content and lead to production of so-called reduced graphene oxide (rGO) [6]. The complete reduction of graphene oxide would lead to a perfect graphene layer as a product; however, there almost always remain some oxygen-containing functional groups, since not all sp^3^ bonds return back to a sp^2^ configuration [11]. By choosing different reducing agents, different carbon to oxygen ratios and different chemical compositions can be achieved in reduced graphene oxide [12]. Robust and flexible graphene oxide flakes offer a large number of opportunities for use in different geometrical forms, such as paper (layers), fibers, or three-dimensional (3D) foams (aerogels) (Figure 2).

Graphene (G) exhibits unique thermal, electrical, and mechanical properties arising from its strictly two-dimensional (2D) structure, and offers immense potential for technical applications. Graphene has a large theoretical specific surface area (2630 m^2^·g^−1^) [7], high intrinsic mobility (200,000 cm^2^·v^−1^·s^−1^) [13], a high Young’s modulus (~1.0 TPa) [14], high thermal conductivity (~5000 Wm^−1^·K^−1^) [15], high optical transmittance (~97.7%) [16], and good electrical conductivity. Graphene-based membranes are impermeable to all gases and liquids (i.e., are vacuum-tight) [17]. The variety of exquisite physicochemical and biological properties listed above shows the potential that graphene-based materials have for applications in many science fields. Indeed, the emergence of graphene-based materials has so far seen their use in a variety of fields that include bio-electronics [18], tissue engineering [19,20,21], drug delivery [2,20], antibacterial materials development [22,23], biosensing [24,25], gene delivery, [1], cancer treatment [26], and other biomedical applications [20,27,28,29,30]. Figure 2 summarizes the most important properties of graphene-based materials that contribute to applications in biomedical fields. The incorporation of graphene-based nano-fillers offers the possibility to tune the mechanical properties of native materials, the possibility to add binding sites for further bio-functionalization with biological molecules, and additional properties; for example, conductivity for regulating cell behaviors, such as cell proliferation and differentiation, which promotes specific tissue regeneration [30,31,32,33].

Aerogels are porous solids systems (solid foams) with a predominance of open pores in which the dispersed phase is a gas [34]. Aerogel structures that have been prepared from graphene can reach record low densities 0.16 mg/cm^3^ [35], which also granted them a special place in the Guinness World Records 2015 [36]. Graphene aerogels of density 0.16 mg/cm^3^ are 7.5 × lighter than air [37], possess 10,000 times higher stiffness than the starting material [38], a lower heat transfer coefficient, and a high specific surface area, and can be easily functionalized for specific substance adsorption, such as oils [39] or nuclear radiation [40]. The porosity of aerogel materials can sometimes be so high that the term “science of empty space” is justified [41]. Typically, aerogels can be prepared from different precursors (e.g., carbon, polymers, silica, metals, metal oxides, quantum dots, composites), which diversifies their applicability, which remains large even when one is only taking into account carbon-based aerogel structures. The applications of aerogels span from drug delivery systems in 2D GO sheets and live cell imaging [42] to tissue engineering [43].

With regard to the extent of the large possibilities of graphene-based structures, we endeavor to give a brief overview of their use in drug delivery for anticancer therapies, in tissue engineering, and as imaging agents. We start by briefly exploring the toxicity of these materials.

## 2. The Toxicity of Graphene-Based Materials

The growing biomedical field of applications of graphene-based materials raises questions about their short- and long-term (cyto)toxicity [44,45]. It is known that the cytotoxicity of G flakes depends on the flake size. Smaller flakes are more cytotoxic and show higher cellular internalization and affect cellular functionality to a greater extent [46]. The number of oxygen functional groups that are attached to the surface also plays an important role [47]. For larger C/O levels, flakes are less cytotoxic, which can be correlated to partially reduced GO structures (rGO) [48]. Wu et al. have shown that cell behavior is strongly responsive to the rGO structure [49]. In particular, they formed few-layer rGO films and controlled the reduction level and surface oxygen content. Their results showed the strong influence of oxidation levels on cellular behavior, with cell attachment, proliferation, and phenotype being best when cells were cultured in proximity to ‘moderately’ reduced GO (mrGO). It was further observed that cell performance decreased significantly with an increased level of thermal reduction.

It is worth mentioning that cytotoxicity was observed to be cell-dependent, e.g., a loss in viability was observed for the human lung adenocarcinoma cell line (A549) and an increase in viability for the human colon cancer (Caco2) and monkey kidney (Vero) cell lines [11,50]. The main tendency is decreasing viability in time, the rate of which is faster for smaller flakes and for higher GO/rGO concentrations [50]. The statement about size seems, surprisingly, to not be valid in case of graphene quantum dots (GQDs) (<10 nm), where the cytotoxicity, when tested on the human osteosarcoma (MG-63) cell line, was low. More generally, the influence on cytotoxicity of size and carbon type can be found in dedicated reviews [51,52,53]. Adding up to 400 mg of GQDs to 150 mL of culture medium (10^4^ cells) did not weaken the cell activity significantly, as shown by an MTT assay [54]. However, the typically used nanotoxicity MTT assays can generate non-specific signals because of the spontaneous reduction of MTT reagent, especially by G and GO [55,56]. Instead, water-soluble WST-based assays have become more widely used and indeed seem to be more appropriate for studying G/GO toxicity versus a variety of cell lines tested in different conditions. For example, Lasocka et al. demonstrated that pristine G monolayers have no cytotoxicity toward murine fibroblasts (L929) as measured by a WST-8 assay and a trypan blue test [57]. Moreover, pristine G increases cell proliferation and causes cells to rearrange the architecture of their cytoskeleton. Furthermore, another group used a WST-1 assay to evaluate the cytotoxicity of thin pristine GO films and those treated for 100 s by NH_3_ plasma [58]. The group observed that the plasma-treated surfaces, which had their surface charge changed to positive, increased the viability of the HeLa cancer cell line obtained as compared to pristine GO, indicating the importance of the surface chemistry of the GO structures. Another group reported that exfoliated pristine G caused a significant decrease in the viability of rat alveolar macrophage cells (NR8383), again measured by a WST-1 assay [59]. Interestingly, the decrease in cell viability was not accompanied by an increase in reactive oxygen species (ROS) generation, in contrast to what others have previously observed [60].

The cytotoxicity aspects of graphene-based materials should not be considered alone. Another important aspect to consider is the degradability of the formed structures, which will also intrinsically depend on the type and size of the used raw material. This issue is still being actively pursued in the field, for example by Mukherjee et al. [61], who showed that GO sheets of differing lateral dimensions were effectively degraded by neutrophils. Moreover, the degradation products of GO were found to be non-cytotoxic and did not elicit any DNA damage in the bronchial epithelial cell line BEAS-2B. Taken together, these studies have shown that neutrophils can digest GO and that the biodegraded GO is non-toxic for human lung cells. Kurapati et al. have also examined the biodegradation of graphene-based materials [62]. They studied the biodegradation of two types of water dispersible G: single-layer (SLG) and few-layer graphene (FLG). The physicochemical properties of SLG and FLG varied not only in terms of the layer number, but also in terms of their surface chemistry due to different preparation methods, which overall were found to affect the degradation process. These results show that G flakes can be degraded either by myeloperoxidase (MPO) secreted by activated neurophils or by recombinant MPO, indicating that pristine G indeed can be degraded by our immune system. These results have large implications on the possible use of this type of material in biomedical applications in vivo, suggesting that the human organism may be able to fully remove the material after it has served its function.

To ultimately reach final applications, graphene-based materials need to be properly assessed in vivo. One of the most basic in vivo studies shows that GO administered at a dose of 0.25 mg via tail-vein injection does not affect the lifespan of mice, while a dose of 0.4 mg is too much and four out of nine mice died within 7 days post-injection. The autopsy showed GO granuloma localized in the lung, liver, spleen, and kidney, but no GO was detected in the brain [63]. Further, it was found that G possesses anticoagulation properties and does not cause red blood cell hemolysis [64]. In another study, the authors injected 440 μg in 200 μL of graphene quantum dots (with a lack of QD size) intravenously to mice, and during in vivo studies a fluorescence emission from the bladder and the urine was detected. Ex vivo studies have shown weak fluorescence in the liver and strong fluorescence in the kidneys [65]. GO exposure to the eye in a rat did not cause acute eye irritation (100 μg/mL); short-term repeated GO exposure generally resulted in reversible damage to the eye via oxidative stress [66]. Most of the current in vivo research on pure-graphene-based materials points to the dead-end route with little progress in comparison to any used controls.

The biocompatibility of pure-graphene-based structures can be further increased by G/GO surface functionalization or coatings. One of the most frequently used polymers is poly(ethylene glycol) (PEG), which has been shown to decrease cytotoxicity both in vitro [67] and in in vivo studies [68]. The surface chemistry oscillates between the affiliates of the GDs family, and even before any surface modification it determines the hydrophilicity or hydrophobicity, stability, and dispersibility of these materials in physiological conditions or in other materials, such as hydrogels [21,69]. Graphene, GO, and rGO elicit toxic effects both in vitro and in vivo, whereas surface modifications can significantly reduce their toxic interactions with living systems [62,70,71]. A detailed analysis of the most recent original research reports along with the earlier review publications unambiguously confirms that graphene in many of its forms and derivatives must be approached as a potentially hazardous material and careful characterization has to be performed [72,73]. In particular, with the current trend of producing larger GO flakes [74], various structures based on GO, such as graphene oxide paper or foam [75,76,77], and when using various graphene derivatives (GDs) based on GO as nano-fillers [21], the exact cytotoxicity will still be of great concern and remains unknown until further studies are performed [74,78]. GO produced via the modified Hummer’s method quite often contains endotoxins that limit its further biomedical potential. Very recently, Parviz and Strano came up with chemical and mechanical protocols for stable, endotoxin-free GO and GD aqueous dispersions production [79]. This could be the rising ‘star’ among new production techniques for fully biocompatible GO flakes, if the scaling-up procedures can be effectively translated to the additive manufacturing level and mass-production capabilities.

**Figure 3:**
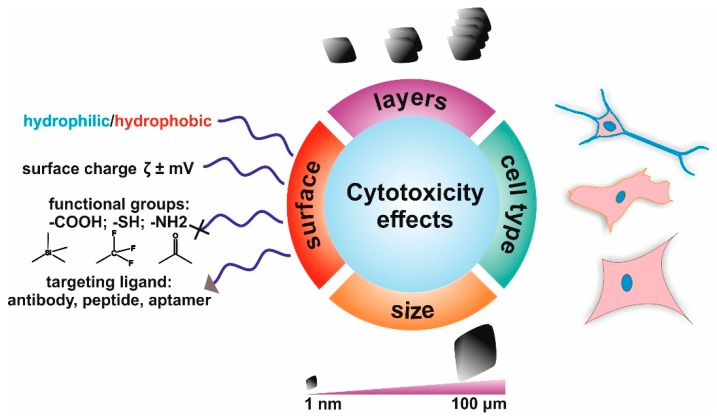
A scheme depicting the typical factors that affect the cytotoxicity of graphene-based materials, including: surface chemistry, physicochemical properties, the number of layers of G/GO/rGO, their size, and the cell type interacting with them.

The aforementioned results emphasize the important role of the surface physicochemical characteristics of graphene and graphene-based materials in their interactions with biomolecules and cells (Figure 3). In particular, the role of their surface chemistry, size, and ability to adsorb active biomolecules has a huge impact on their cytotoxic effects and should be presented in all manuscripts concerning the use of such materials for biomedical applications [71,80].

## 3. Graphene-Based Materials in Anticancer Drug Delivery Systems

Graphene (G) is highly hydrophobic, whereas GO is decorated by oxygen-containing hydrophilic groups [21]. This unique surface chemistry allows for π–π stacking interactions and electrostatic interaction to occur with other molecules in its vicinity. This allows for both physical and chemical binding of drugs to the surface of G/GO for drug delivery applications [81]. Ever since Liu et al. utilized PEGylated GO to deliver a camptothecin (CPT) analogue [82], G and GO have seen a rapid increase in their use as vehicles to deliver drugs, including antibiotics, peptides, antibodies, genes, poorly soluble drugs, and anticancer drugs. In this section, we briefly discuss these delivery mechanisms by means of G and GO with emphasis on using G/GO in anticancer therapies amongst other fields.

An ageing population, smoking, stress, and a lack of physical activity are major contributors to the rapid increase in the cancer mortality rates of many types of tumors [83]. It remains challenging to achieve proper anticancer treatment due to factors such as low bioavailability and poor targeting of chemotherapeutics [83]. G and GO have been extensively explored as drug carriers, biomolecule sensors, and cellular imaging agents in anticancer therapies [26]. The simplest strategy is fabrication of a nanocomposite/hybrid built from GO and drugs. In one case, GO and chlorogenic acid (CA) were presented as a pH-sensitive platform for the slow release of CA from GO [84]. Both the drug itself, a carrier, and their nanocomposite showed a negligible toxic effect toward a normal cell line, while a highly cytotoxic response was observed for cancer cell lines [84]. In another example, GO was chemically functionalized with amino groups and combined with carboxymethyl cellulose as an anticancer system with a controlled and targeted release of the Doxorubicin (Dox) drug [85]. Recently, Li et al. reported that folic acid (FA) combined with polyethyleneimine (PEI)-functionalized GO was used as a carrier for two new copper complexes toward the nasopharyngeal carcinoma cell line (HNE-1) [86]. The developed FA/PEI/GO exhibited good water solubility and biocompatibility, and in vitro cytotoxicity studies show that copper complexes have a good inhibition effect on the carcinoma cell line. These nanocarriers allow for sustained drug release, targeting inhibition and late stage apoptosis of HNE-1 FA positive+ cells, which can reduce side effects during chemotherapy [86]. 

Another strategy that is utilized in cancer treatment is the formation of injectable hydrogels with GO-based nanofillers that can bind and subsequently release typical hydrophobic anticancer drugs [87]. One example is G/GO composite supramolecular hydrogels that incorporate Camptothecin (CPT) and Doxorubicin (DXR) [88]. These hydrogels were able to release anticancer drugs more slowly than Pluronic F-127 solution due to the higher binding affinity of hydrophobic drugs to the G/GO present in the gels, which offered an opportunity for controlled release [88]. In another study, thermoresponsive poly(*N*-isopropylacrylamide) (PNIPAM) was used as a polymer matrix for GO nanosheets, which resulted in a hybrid, self-healable, supramolecular hydrogel [89]. Subsequently, Dox was encapsulated in the formed hydrogels and its release from the hybrid hydrogels resulted in the death of most of the human cervical cancer cells (HeLa) after 48 h. T. Kavinkumar et al. [90] prepared rGO–silver nanoparticles (rGO-AgNP) composites by a simple, fast, nontoxic, and eco-friendly approach. These materials showed pronounced anticancer affinity towards human A549 lung cancer in a dose of 30 µg/mL, and the authors confirmed that they work significantly better than the controls (GO, rGO, and GO-AgNP). Moreover, it was observed that rGO-AgNP induced increased production of free radicals (ROS) in A549 lung cancer cells that resulted in a free radical attack on membrane phospholipids that lead to cell death through apoptosis. Another study demonstrated that rGO-AgNP has great cytotoxic potential in different subpopulations of human ovarian cancer stem cells (OvSCs), especially in ALDH^+^CD133^+^ cells, which are characterized by high tumorigenicity [91]. These composites significantly reduced the number of OvSCs colonies, and enhanced the expression of pro-apoptotic genes while simultaneously downregulating the anti-apoptotic gene *Bxl-2* [91].

Functionalized GO chips for the highly sensitive capture of circulating tumor cells (CTCs) in the blood of cancer patients were also presented in another study [92]. CTCs are responsible for the spread of cancer to secondary sites leading to the development of metastases, which are a major cause of mortality in cancer patients. It remains crucial to isolate these cells in order to prevent tumor metastasis. One of the effective methods to isolate CTCs with high sensitivity and low target cell concentration from the blood of pancreatic, breast, and lung cancer patients was actually developed by the use of phospholipid–polyethyleneglycol-amine (PL–PEG–NH_2_) functionalized GO nanosheets on a patterned gold surface. Interestingly, the authors observed that the same chip without the GO did not yield the same positive results; however, no discussion about the importance of GO was given. Nevertheless, such studies demonstrate a significant accomplishment towards the development of graphene-based diagnostic chips that can be used as sensors for targeting biomolecules from cancer patients’ samples, eventually leading to cheap cancer sensors.

Graphene quantum dots (GQDs) are another form of G used as carriers for cancer drug delivery applications. In one of the studies, GQDs were functionalized by a commonly used arginylglycylaspartic acid (RGD) peptide sequence, which binds to the “αvβ3” integrin site in a cancer cell. When Dox drugs were loaded onto the GQDs-RGD, their enhanced uptake by the PC-3 and DU-145 human prostate cell lines was observed in comparison to pure Dox [93]. Ko et al. presented another approach in which (Herceptin)-labelled GQD-based nanocarriers (GQD-comp) were used in the treatment of Human Epithelial Growth Factor Receptor 2 positive v+ (HER2) breast cancer [94]. These GQD-comp were also loaded with Dox, which was released in a specific temperature and pH environment, which eventually led to inhibition of the proliferation of human breast cancer cells.

Overall, many multimodal approaches that combine G/GO structures have been proposed for anticancer therapies. However, we still lack the full in vivo characteristics of the proposed G/GO nanomaterials, and it remains a challenge to assess their biodistribution, biodegradability, and the targeted effects in many types of cancers. Future work in the field will need to focus not only on multimodal approaches but mainly on the applications and feasible sites. In particular, all proposed structures will need to explicitly prove their safety in extensive trials prior to their proper use. Taking into consideration the fact that these materials could one day save someone’s life, their purity, firstly, will need to be of the highest level. To do so, the characterization techniques will need to be properly standardized and regulations will need to ensure that there are proper definitions for the materials and their properties.

## 4. Graphene and Graphene Oxide in Tissue Engineering

### 4.1. Two-Dimensional (2D) Substrates

Engler et al. revolutionized the tissue engineering field in 2006 by showing explicitly the effect of substrate stiffness on the differentiation of mesenchymal stem cells (MSCs) into different cell lineages [95]. They achieved that by preparing three polyacrylamide matrices with a Young’s modulus of 0.1–1 kPa, 8–17 kPa, and 25–40 kPa, respectively, which induced the differentiation of stem cells into the brain, muscle, and bone lineages, respectively, for the first time, suggesting that cell differentiation is highly dependent on the stiffness of the substrate material [95]. The high Young’s modulus of graphene (~1 TPa) and its specific shape can, therefore, offer a way of forming composite materials with tailored mechanical properties for inducing a specific biological response [14]. In fact, graphene-based materials of varying mechanical properties have been explored for wound healing [22,96], stem cell engineering [30,97,98,99], and regenerative medicine and tissue engineering [20,100,101]. The excellent mechanical properties of graphene (high elasticity, strength, flexibility) and the ability to tailor various functionalities on flat surfaces [14] make graphene a potential reinforcement material in hydrogels [21], biodegradable films [102], electrospun fibers [96], and other tissue engineering scaffolds [103]. For example, the incorporation of GO into polyvinyl acetate (PVA)-based hydrogels significantly enhanced the tensile strength (132%) and the compressive strength (36%) of composite hydrogel soft solids without affecting the cytotoxicity toward osteoblast cells [104]. As another example, G-reinforced chitosan films showed enhanced stiffness and, again, the toxicity of the structure was not compromised on a murine fibrosarcoma L929 cell culture [102].

One of the interesting approaches for forming composite GO structures is their covalent crosslinking with biopolymers. One such approach was the formation of GO–chitosan hydrogel scaffolds that were prepared by covalent linkage of chitosan amino groups with carboxylate groups of GO [103]. These GO–chitosan hydrogels exhibited a significant improvement in pre-osteoblast MC3T3-E1 cells’ adhesion, differentiation, proliferation, and calcium phosphate deposition. Although G sheets are non-biodegradable materials, the low G content in G/chitosan composites may limit any possible negative influence of G on cells after chitosan has decomposed in the body, as was clearly evidenced by the cytotoxic measurements that were presented in the previous section.

Currently, tissue damage remains one of the most crucial aspects that contributes to human death. In this regard, a number of studies have been performed that explore the use of graphene for stem cell engineering and musculoskeletal tissue engineering [30,97,98,99,105]. One such study is by Chen et al., who investigated the effect of G and GO platforms for the proliferation and differentiation of induced pluripotent stem cells (iPSCs) [97]. The authors observed that pure graphene surfaces support iPSC cultures and allow for their spontaneous differentiation. As compared to glass, GO showed faster iPSC proliferation and endodermal differentiation, whereas G exhibited proliferation comparable to glass and suppressed the endodermal differentiation. One can conclude from this work that GO-coated scaffolds may be used to direct iPSC differentiation into endodermal lineages (hepatocytes and insulin-producing β-cells), whereas G-coated surfaces can be used for subcultures and the expansion of iPSCs as, interestingly, this maintains their pluripotency. G-coated surfaces with varying stiffness and roughness have also been evaluated for the differentiation of human mesenchymal stem cells (hMSCs) and pre-osteoblasts into osteoblasts [30]. G- and GO-coated surfaces exhibited accelerated cell adhesion, proliferation, and differentiation of hMSCs as compared to those cultured on polydimethylsiloxane (PDMS), polyethylene terephthalate (PET), glass, and Si/SiO_2_ substrates [30]. One of the most interesting aspects of further work with hMSCs was that G remarkably accelerated their differentiation in vivo in a rat, which was comparable to the presence of bone morphogenic protein-2 (BMP-2) on uncoated surfaces [98].

Human-adipose-derived stem cells (hASCs) have also been extensively studied in tissue engineering due to their being easy to obtain from patients. Chung et al. have investigated the effects of GO-coated substrates on the fate of hASCs [106]. As others have already shown, the nanoscale topography of artificial substrates does greatly influence the fate of stem cells, including adhesion, proliferation, and differentiation [107]. With this in mind, Chung et al. introduced a GO film as an effective platform for controlling the function of hASCs. To form thin GO films, GO was deposited on a glass substrate using a layer-by-layer self-assembly method, where the substrate was simply immersed in the GO solution (2 mg/mL). The hASCs grown on the GO films showed increased adhesion, which was indicated by a large number of focal adhesions, and a higher correlation between the orientations of the actin filaments and the vinculin bands compared to hASCs grown on the glass (uncoated GO) substrate. It was also found that the hASCs showed a stronger affinity toward GO films than the glass. Interestingly, the GO film enhanced the differentiation of hASCs, including osteogenesis, adipogenesis, and epithelial genesis, while the chondrogenic differentiation of hASCs was decreased when compared to a control substrate (tissue culture polystyrene plates).

Graphene-based materials were also explored in musculoskeletal tissue engineering using mouse myoblast C2C12 cell lines [105]. The oxygen content on the GO was shown to be crucial and induce a higher myotube fusion/maturation index and upregulated expression of myogenic genes (MyoD, myogenin, troponin T, and myosin heavy chain) when compared to the rGO. The basic difference between GO and rGO is the affinity for protein adsorption, such as serum proteins, and this is strongly linked to gene regulation. In particular, the adsorbed proteins are believed to cause enhanced cellular behavior when present on G derivatives in comparison to their free form in the solution. The affinity of G for biomolecules adsorption was also explored by Nayak et al., who used bone morphogenetic protein 2 (BMP-2) adsorbed on G as a promising biocompatible scaffold that does not hamper the proliferation of human mesenchymal stem cells (hMSCs) and accelerates their specific differentiation into bone cells [108]. The differentiation rate was comparable to the one achieved with common growth factors, demonstrating the potential of G in stem cell research. In another study, fully fluorinated G induced a higher proliferation of MSCs promoting neuronal differentiation in comparison to non-fluorinated G [99]. This was further enhanced when MSCs were confined into micro-channels patterned onto fluorinated graphene in the absence of any chemical stimulants [99]. Wang et al. have also looked at the influence of chemically functionalized carboxylated GO with different surface charge groups (amino- (–NH_2_), poly-m-aminobenzene sulfonic acid- (–NH_2_/–SO_3_H), and methoxyl- (–OCH_3_)) on neurite outgrowth and branching [109]. Surprisingly, positively charged GO with a ζ-potential value of 40.4 mV (functionalized with NH_2_ groups) was found to be more beneficial for neurite outgrowth and branching.

Overall, the abovementioned studies highlight the importance of understanding the physical interactions in biomaterials and substrates for biological purposes and emphasize the possibilities of modulating biological responses for tissue engineering by both chemical and physical approaches. Even though there exist a large number of studies on the effect of graphene-based materials, the exact elucidation mechanisms, due to the many combinations of protein–G/GO interactions or the endless chemical functionalization of G/GO, are yet to be revealed. Perhaps the growing power of artificial intelligence and multi-algorithm-based approaches will soon be able to screen the whole library of cell-influencing factors simultaneously to generate ideal tissue engineering platforms for regenerative medicine.

### 4.2. Beyond 2D

Two-dimensional (2D) graphene-based material coatings on substrates and layered materials are insufficient for tissue engineering, and these need to be further developed in order to mimic a proper, functional 3D extracellular matrix (ECM) environment. Indeed, recent advances in this field include the exploitation of graphene foams (GFs) as three-dimensional (3D) scaffolds for a neural stem cell (NSC) culture [27] and human stem cell differentiation [30]. Three-dimensional GFs support the attachment and viability of hMSCs, and induce spontaneous osteogenic differentiation [30], which makes possible the development of graphene-based strategies for osteogenic and conductive tissue-engineered scaffolds. The fabrication of 3D GFs at a low cost was based on a Nickel (Ni) foam precursor and was found to be highly scalable to larger sizes that could be used to replace, for example, bones. Indeed, these 3D GFs were shown to act as robust scaffolds for an NSC culture in vitro, where NSC growth was strongly supported and cells were kept in a more active proliferation state with an upregulation of Ki67 expression when compared to 2D graphene films [27]. Furthermore 3D GFs enhanced the NSC differentiation towards astrocytes and neurons and were shown to be an efficient conductive platform to mediate electrical stimulation for differentiated NSCs [27]. Likewise, culturing readily available hMSCs in these 3D constructs, along with their potential for multilineage differentiation, holds great promise for novel, advanced strategies in regenerative medicine. Whilst the efficiency of the scaffolds is promising, the incorporation of Ni alloys into the human body may have potential side effects, and their biodegradability is compromised. Therefore, others have focused on more bio-friendly approaches to 3D G/GO-based structures. For example, Shin et al. fabricated 3D composite scaffolds using gelatin methacrylate (GelMa) and GO [110]. The incorporation of GO into GelMa hydrogels enhanced their mechanical and electrical properties with no adverse effects on encapsulated fibroblast cells [110], which highlights the potential of the use of GO as a nanofiller in hydrogels for 3D cell culture growth applications and the engineering of functional tissue constructs. The selection of graphene-based materials was also encapsulated in a series of peptide-based hydrogels, and was shown to support the viability of hMSCs [21]. The playground between the molecules that form the hydrogel network and functionalized G/GO materials offers infinite opportunities for the fabrication of 3D scaffolds with the desired characteristics for 3D tissue engineering constructs.

**Figure 4:**
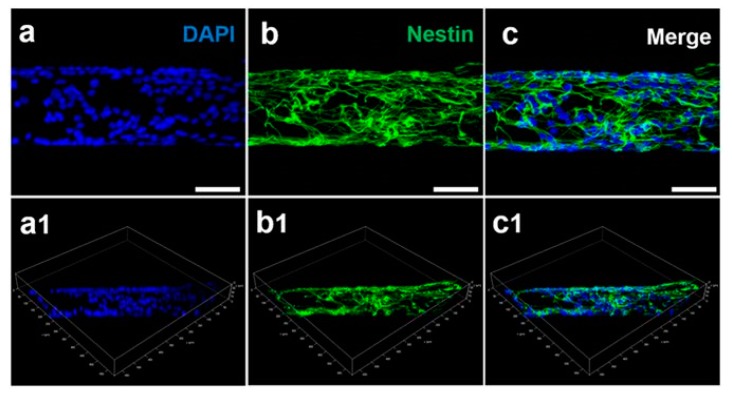
Neural stem cell (NSC) adhesion on a nanostructured rGO microfiber. Two-dimensional confocal laser scanning microscopy fluorescence micrographs of NSCs that have proliferated on a nanostructured rGO microfiber for 5 days; immunostaining makers were DAPI (blue) (**a**) for nuclei and Nestin (green) (**b**) for NSCs, and their images were merged (**c**). (Scale bar = 50 μm). The 3D structure CLSM fluorescence micrographs of the nanostructured rGO microfiber with DAPI- (**a1**) and Nestin- (**b1**) stained NSCs are also presented; (**c1**) is the merged micrograph. The unmodified text and graph have been reproduced under a Creative Commons Attribution 4.0 International License from [113].

Another approach in tissue engineering is to use pure 3D G foams that are produced via metal-free methods [111]. Pure G foam was examined as an electrically conductive scaffold for testing the effects of the electrical stimulation of human neural progenitor cells (hNPCs) that were derived from a patient’s fibroblasts that were available in the cell bank. The authors demonstrated that the hNPCs adhered to the scaffold and formed a neural network over the course of the first week. Cells after electrical stimulation were found to have larger average soma than cells without any electrical stimulation. Electrical stimulation of hNPCs cultured on a 3D G scaffold caused an increase in their differentiation and maturation into neurons [111]. GFs are also efficiently used to form regeneration tubes for neural tissue engineering [112]. For instance, GO foam (GOFs) layers were rolled to obtain 3D cylinder-like scaffolds. These were then UV irradiated and partially deoxygenated to form electrically conductive scaffolds. Under electrical stimulation of GOFs, human neural stem cells (hNSCs) grew directly in the direction of the main axis of the scaffolds. Electrically stimulated hNSCs cultured over 2 weeks on rolled GOFs extensively proliferated and more efficiently differentiated into neurons as compared to glial cells, which indicated promising results that could be useful in regenerative medicine for the nervous system [112]. The effect of rGO that was shaped into 3D porous wires that were synthesized through a capillary hydrothermal method on the neural differentiation of neural stem cells (NSCs) was also evaluated [113]. These findings indicated that the flexible, mechanically strong, nanoporous, biodegradable, and biocompatible nanostructured rGO microfibers not only offered a more powerful substrate for NSC adhesion and proliferation when compared with 2D G films and tissue culture plastic dishes, but also regulated the NSC differentiation into neurons that formed a dense neural network surrounding the microfiber (Figure 4).

Another approach is to use hybrid 3D GFs with additional coatings. One such case was reported in which a commercially available hydrophobic pristine 3D GF was coated by a well-established biocompatible collagen that was cross-linked with genipin to form a hybrid 3D GF porous, hydrophilic, and conductive scaffold for the culture and differentiation of mouse mesenchymal stem cells [114]. The obtained materials were found to be non-cytotoxic to mouse MSCs and promoted the expression of β-tubulin, neural nuclear antigen (NeuN), and tyrosine hydroxylase (TH), which caused extensive neurite elongation and differentiation into dopaminergic (DA) neurons [114]. In another approach, 3D rGO aerogels were functionalized by chitosan and mineralized by an incubation with simulated body fluids (SBF) to mimic the formation of natural bone [43]. The obtained chitosan rGO aerogels, which consisted of self-assembled, micrometer-sized rGO sheets, indeed promoted osteogenic differentiation, matrix formation, and higher viability of the osteoblast-like cell line (MG63) in comparison to non-mineralized, chitosan-functionalized aerogels [43].

Obviously, 3D structures that mimic the tissue’s extracellular matrix are highly desirable in the tissue engineering field. Due to their intrinsic structural nature (very high aspect ratios), G and GO have so far mainly been used as coatings and substrates of flat surfaces and materials. However, there is a large trend to scale the results to the 3D world, either by simply incorporating the 2D flakes in a 3D biopolymer matrix or by forming hybrid foam-like structures that can better mimic the desired properties and characteristics of the chosen tissue. We envisage the use of such materials in real-world applications and wait for the future outlook, which will most likely consist of changes to the mass production of large quantities due to the increasing use of additive manufacturing techniques. In particular, we see a large rise in the 3D printing of graphene-based inks that, in the near future, may replace the complex 3D structure of tissues with enhanced properties [31,115,116,117].

## 5. Graphene-Based Materials in Bio-Imaging

Bio-imaging, which focuses on imaging selected biological entities, remains a crucial aspect of regenerative medicine [118]. Imaging techniques that utilize graphene-based structures include fluorescence/confocal imaging [119], surface-enhanced Raman scattering (SERS) [120], coherent anti-Stokes Raman scattering imaging (CARS) [121,122,123], magnetic resonance imaging (MRI) [124], positron-emission tomography (PET) [125,126], ultrasound imaging [127,128], photoacoustic imaging [129], and electron paramagnetic resonance imaging (EPRI) [130,131]. These can lead to a greater understanding and monitoring of multiple processes in living cells, tissues, and the whole body. Using bio-imaging, one can track the development of abnormal processes, such as cancer development, hypoxia/hyperoxia, or necrosis. Indeed, the two basic requirements in bio-imaging include: (1) fast and sensitive detection tools (technological equipment), and (2) effective contrast agents in the form of (nano)materials that will possess all biological requirements, i.e., biodegradability, biocompatibility, and crossing of the blood–brain barrier (BBB) if required, or an appropriate diffusion time/mechanism of active transport to the diseased/targeted tissue, high specificity and sensitivity, and high applicability.

The most often used imaging technique involves fluorescence. This has become the gold standard technique that is used in biology and regenerative medicine. In particular, Forster Resonance Energy Transfer (FRET) imaging [132] is most common subtype of fluorescent imaging, which allows obtaining the highest image spatial resolution, exceeding the inherent diffraction limit (~λ/2) of conventional optical microscopy. In this imaging method, fluorescence signals can be generated that are sensitive to molecular conformation, association, and separation in the 1–10 nm range. Here, two dyes are used: the first one is called a donor, which absorbs the external laser energy and then further transfers it to the second molecule, which is called an acceptor, where the final fluorescence detection process occurs.

Some techniques, including computer tomography (CT), PET and MRI, and ultrasound imaging are already well-established and can be used in human studies. The EPRI technique is a relatively new technique under strong development. By using this method, one is able to detect and quantify multiple biological parameters in a tumor microenvironment in vivo in small animal models, including: pO_2_ [133,134,135,136], pH [131,137], and redox status [131,138]. Usually, in this method, triphenylmethyl radical (TAM) derivatives [139] or other radicals [140] are used; however, it was also found that carbon-black-based ink is also a suitable medium used in EPRI for localized oxygenation sensing [141]. Raman spectroscopy imaging is possible via mapping of the functional chemical vibrations. It is, however, limited only to cell cultures and small-sized tissues (ex-vivo) due to the limited laser light penetration (~1 µm) into the human (or animal) body and the lower signal sensitivity in comparison to the CARS imaging technique. CARS offers a higher signal sensitivity, which further allows measurements to be taken in cell cultures [142], ex vivo tissues, and, recently, in vivo in mice with a sub-micrometer resolution [143].

Structures such as G, GO, and the related G-based composites that are used in bio-imaging are usually treated as platforms for the modification of existing contrast agents [144,145]. However, these structures also offer metal-free approaches that typically possess high cytotoxicity [146]. The most frequently used G/GO-based sensor is used in fluorescence imaging [147]. G/GO-based substrates are typically transformed into carbon/G quantum dots (QD) that consist of ultra-small flakes ~10 nm in size, which exhibit size-dependent and surface-chemistry-dependent fluorescence (a quantum yield of 11.4% [54]). A further increase in the biocompatibility of such structures can be achieved by surface functionalization. The most common biopolymers that are used for that purpose are: polyethylene glycol (PEG) [67,148,149,150], polypeptides [151], polyethyleneimine [152], and polystyrene [153].

The next and simultaneously higher level of bio-imaging development is the fabrication of multimodal materials (nanoparticles) that can simultaneously be used in more than one way, e.g., in photothermal and photodynamic therapy and tracking (imaging) [154,155,156,157]. An example is a rGO/iron oxide NP covered with PEG (rGO–IONP–PEG), which can be applied in vivo for photothermal therapy (PTT) and as a triple-mode sensor: fluorescence, photoacoustic tomography (PAT), and magnetic resonance (MR) imaging [158,159]. Another example is fluorinated GO as a magnetically responsive drug carrier with the possibility for imaging via magnetic resonance imaging (MRI) and photoacoustic tomography [160]. Lin et al. have recently summarized the versatile imaging capabilities of graphene-based nanomaterials in their work and came to the conclusion that, by combining other materials with specific properties of GOs, multimodal imaging can be achieved in a single platform [161]. We anticipate that G and GO-based materials and their various structured materials will play a crucial role in the next decade in imaging in regenerative medicine and evaluating the fate of therapeutics in vivo.

## 6. Conclusions

Graphene-based materials in the form of flakes, layers, foams, nanofillers, nanodots, and other structured materials have had a profound impact on the regenerative medicine and biomedical fields. The cytotoxicity of these materials is strongly dependent on the intrinsic flake sizes that are used in the final material, the structure and shape of the material, its surface chemistry, and the type of cultured cells. The fundamental interactions between the surface and surface edges of materials with the biomolecules, drugs, and cells are driving the biological pathways, in many cases in still-unknown ways. The toxicity aspects of the structure of graphene/graphene oxide are to this day debatable and remain of high interest to the scientific community. Likewise, the structure of graphene/graphene oxide-based materials influences their possible application fields, which were briefly reviewed here and include anticancer drug delivery therapies, tissue engineering, and multimodal bio-imaging.

Almost all of the drug delivery anticancer approaches rely on the successful adsorption of the drug on the surface of the graphene/graphene oxide structure. Many efforts have been focused on the use of a well-established anticancer drug, such as doxorubicin (Dox), and the use of versatile graphene-based and graphene-based hybrid vehicles for its delivery or co-delivery and imaging. However, to this day, the in vivo work has shown virtually no improvement in using such vehicles in comparison with pure drugs, or there have been no attempts to perform in vivo evaluations. This shows a challenging problem with anticancer therapeutics where the drug/material’s biodistribution, biodegradability, and the targeted effects are still unknown. Moreover, little has been done to derive new drugs using graphene-based materials as platforms. We see particular promise in the graphene quantum dots that have been used in multimodal therapies, which may, simultaneously, have selective sensitivity, be effective contrast agents for fluorescence imaging with a high quantum yield, and be used for photothermal and photodynamic therapy. Indeed, small doses of materials with proper characteristics were shown to be acceptable for cell culture growth and bio-imaging in terms of the cytotoxic effects.

Interestingly, graphene-based materials remain highly successful in the tissue engineering field for the regeneration of a variety of tissues. However, most of the successful work has been performed on 2D substrate systems, which could prove that G/GO-based flake coatings are useful for various biomedical devices. On the other hand, G/GO have been used as nanofillers in a variety of hydrogels or soft-matter materials that add a desired functionality to the native materials, such as the possibility of adsorption for certain proteins or an electrically active percolation network and/or surface that triggers cell proliferation, adhesion, or differentiation. There is a lot of work that focuses these days on using G/GO in 3D systems, either as a pure network or as a hybrid with other materials, which holds great promise for the use of these materials in real-world biomedical applications. Based on the literature survey presented in this review, we believe that there remain many crucial aspects to consider before this field can progress further, namely: nanomaterial purity and characterization and proper definitions to ensure data reproducibility and appropriate data correlation.
